# Highly conductive vertically aligned molybdenum nanowalls and their field emission property

**DOI:** 10.1186/1556-276X-7-463

**Published:** 2012-08-17

**Authors:** Yan Shen, Shaozhi Deng, Yu Zhang, Fei Liu, Jun Chen, Ningsheng Xu

**Affiliations:** 1 State Key Laboratory of Optoelectronic Materials and Technologies, Sun Yat-sen University, Guangzhou 510275, People's Republic of China; 2 Guangdong Province Key Laboratory of Display Material and Technology, Sun Yat-sen University, Guangzhou 510275, People's Republic of China; 3 School of Physics and Engineering, Sun Yat-sen University, Guangzhou, 510275, People's Republic of China

**Keywords:** Molybdenum nanowalls, Thermal vapor deposition, Growth mechanism, Field emission, Electrical conductivity

## Abstract

We report that vertically aligned molybdenum (Mo) nanowalls can grow on various substrates by simple thermal vapor deposition. Individual nanowalls have a typical thickness of about 50 nm and very good conductivity with a typical average value of about 1.97 × 10^4^ Ω^−1^ cm^−1^, i.e., only an order of magnitude less than the value of bulk Mo. The formation process is characterized in detail, and it is found that Mo nanowalls grow from nanorods through nanotrees. The atomic arrangement, lattice mismatch relationship, and competition growth are all believed to contribute to the growth mechanism. The field emission performance is attractive, typically with a very low fluctuation of about approximately 1.18% at a high current density level of 10 mA/cm^2^, and a sustainably stable very large current density of approximately 57.5 mA/cm^2^ was recorded. These indicate that the Mo nanowall is a potential candidate as a cold cathode for application in vacuum electron devices, which demand both a high current and high current density.

## Background

Field emission cold cathode has many important applications [1]. There are typically two areas which have attracted the attention of researchers, i.e., the field emission flat panel display (FED) and the vacuum electron devices, such as X-ray sources [2,3] and microwave sources [4,5]. FED requires large-area cold cathode arrays, which are addressable and thus need low-field emitters. The vacuum electron devices generally require both a high current and high current density. Now, it has been shown that nanostructured materials can give low-field cold electron emission [1]. However, one has come across a number of problems in pursuing a high-current and high-current density cold cathode. The major problem is that there are high-resistance regions in the individual nanostructure using nanowire as an emitter, which can lead to vacuum breakdown when it undergoes a high-current density operation [6]. Even more, so far, the conductivity of many nanotubes and nanowires is not high enough, except that for carbon nanotubes. Carbon nanotubes, however, are easily oxidized during the vacuum sealing process and thus lose its good properties.

We have been trying two other approaches aimed at overcoming the above difficulties. One is to grow nanowires or nanowalls with a high electrical conductivity [7-16]. In the recent years, one has seen a large number of studies focused on the preparation of one-dimensional molybdenum oxide nanostructures, such as MoO_2_ nanorods [7], pinaster-like MoO_2_ nanoarrays [8], and MoO_2_ nanostars [9], with a few cases on two-dimensional (2D) molybdenum oxide nanostructures such as MoO_3_ nanobelts [10]. Our group has been developing a thermal vapor deposition method for synthesizing vertically well-aligned molybdenum oxide nanowires [11-14] and for a large-area preparation of 2D MoO_3_ microbelts [15,16]. In this paper, we report that uniform Mo nanowalls can be grown on various substrates by simple thermal vapor deposition. The morphology and crystalline structure of the nanowalls are characterized by scanning electron microscopy, X-ray diffraction, and transmission electron microscopy techniques. The growth mechanism is discussed. The field emission performance and electrical transport property of the nanowalls are shown to be promising in meeting the requirements of high current and high current density of some vacuum electron devices.

## Methods

The basic thermal vapor deposition method we used in the present study has been described in our previous report [14]. The method is developed based on the chemical reactions observed in the formation of Mo nanowires, which is similar to the formation of tungsten (W) [17]:

(1) $$Mo\left(s\right)+O_{2}\left(v\right)\to MoO_{2}\left(v\right),\Delta G=-69kJ/\left(mol\;K\right)\left(T=1623K\right)$$

(2) $$3MoO_{2}\left(v\right)\to Mo\left(s\right)+2MoO_{3}\left(v\right),\Delta G=-341kJ/\left(mol\;K\right)\left(T=1523K\right)$$

Mo atoms of the Mo boat react with residual oxygen in the vacuum chamber spontaneously to give rise to MoO_2_ (Equation 1). Then, Mo atoms coming from the decomposition of MoO_2_ deposit during a condensation process (Equation 2). Here, we should note that when the substrate temperature is over 1,428 K, the condensation process of MoO_2_ will be difficult. So, the key to synthesize the molybdenum nanostructures rather than oxide ones is the high-enough reaction temperature. Silicon, stainless steel, or silicon carbide was used as substrates in this study. They were first washed with acetone and then with alcohol, respectively, for more than 15 min in an ultrasonic bath. The substrate and Mo boat were then placed in the center of vacuum chamber, keeping them about 5 mm away from each other. The Mo boat as evaporation source was heated by electric current. The vacuum chamber was evacuated using a mechanical pump. When the vacuum reached below approximately 6 × 10^−2^ Torr, high-purity argon (Ar) gas (99.99%) was introduced into the system at a flow rate of approximately 200 sccm, accompanied by high-purity hydrogen (H_2_) gas (99.99%) at a flow rate of approximately 80 sccm. The boat temperature was then increased gradually at a rate of 50 K/min to above 1,623 K, in order to ensure the growth of the metallic molybdenum nanowall rather than the oxide one. After deposition for more than 10 min, the preparation of molybdenum nanowalls was completed. No catalysts were used in the whole process of growth.

It is significant that to obtain molybdenum nanowalls, the substrates should be placed below the Mo boat with a certain distance away, instead of being directly placed inside the boat, as we did when growing Mo nanowires [14]. In the present study, the growth orientation and the driving force of nucleation can be controlled by adjusting the spacing between the Mo boat and substrates.

Field emission scanning electron microscopy (XL-SFEG SEM, FEI Co., Hillsboro, OR, USA) was employed to investigate the morphology of molybdenum nanowalls. Transmission electron microscopy (Tecnai-20 TEM, Philips, FEI Co., Hillsboro, OR, USA), and X-ray diffraction (XRD; Rigaku RINT 2400, Rigaku Corporation, Tokyo, Japan) were applied to study their crystalline structures. The field emission properties of molybdenum nanowall films were measured in the field emission analysis system. In addition, the electrical transport measurement of the individual nanowalls was carried out using a micro-point anode *in situ* in a modified SEM system (JEOL-6380, JEOL Ltd., Akishima, Tokyo, Japan); the details of which are described in our previous work [6].

## Results and discussion

**Figure 1:**
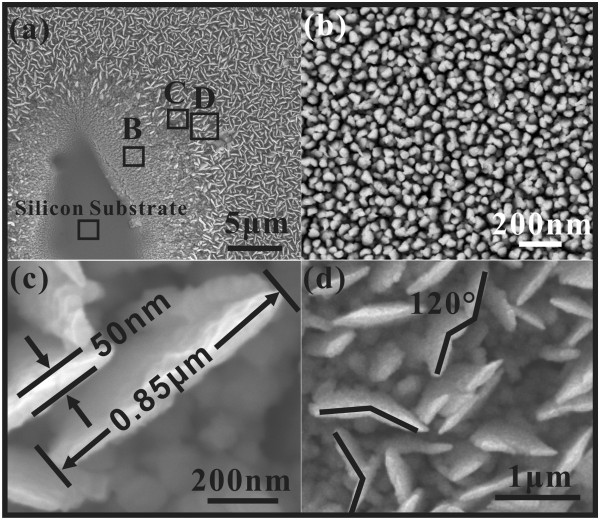
**Top-view SEM images of molybdenum nanowalls film.** The scratched area was deliberately used for analysis. (**a**) Low-magnification and (**b**, **c**, **d**) high-magnification SEM images of rectangular enclosed areas B, C and D in (a), respectively.

Figure 1a shows the top-view SEM image of molybdenum nanowalls grown on the silicon substrate with an artificially scratched area on the bottom left corner. The molybdenum nanowalls are seen to be well distributed all over the substrate, which helps to ensure the uniformity of field emission. The density of the nanowalls is about 3 × 10^8^/cm^2^. Moreover, we can see from the scratched area (Figure 1b) that there is a layer of nanorods existing between the molybdenum nanowalls and underneath the substrate; here, we call it mid-layer. Figure 1c shows that, typically, the width of the nanowall ranges from 0.5 to 1 μm, with their thickness being about 50 nm. Also, Figure 1d reveals that some of the nanowalls have zigzag shapes, usually with an angle of about 120° between adjacent parts.

**Figure 2:**
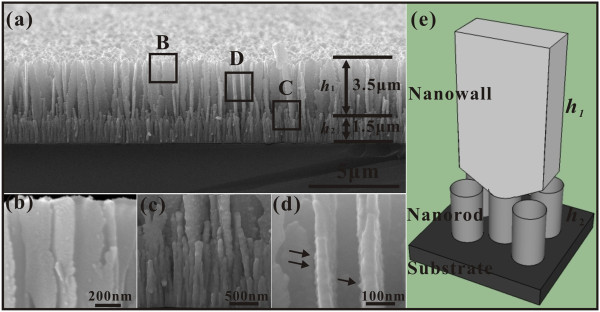
**Side-view SEM images of molybdenum nanowall films.** (**a**) Low-magnification and (**b**, **c**, **d**) high-magnification SEM images of rectangular enclosed areas B, C, and D in (**a**), respectively. (**e**) The corresponding 3D geometrical model of an individual Mo nanowall.

Figure 2 gives the side-view SEM images. From Figure 2a, the molybdenum nanowalls have an average height of about 3.5 μm, and the mid-layer has an average height of about 1.5 μm. From the high-magnification SEM images (Figure 2b,c,d) of the rectangular enclosed areas B, C, and D, respectively, one can see that the nanorods are the roots of the nanowalls (Figure 2c) while these nanorods form the mid-layer. On the other hand, from Figure 2d, one can see that the surface of the nanowalls is typically not smooth. The black arrowheads point out the positions the protrusions on the surface. Figure 2e shows the corresponding three-dimensional (3D) geometrical model of the individual Mo nanowall, as one may intuitively see the structure of such material.

**Figure 3:**
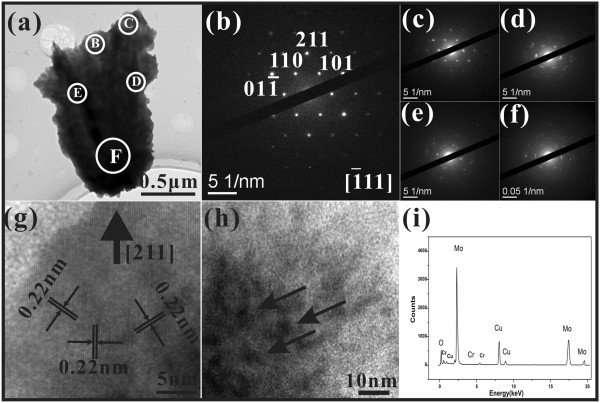
**TEM and HRTEM images, SAED patterns, and EDX spectrum.** (**a**) Typical TEM image of a typical molybdenum nanowall. (**b**, **c**, **d**, **e**, **f**) SAED patterns of circular enclosed areas B, C, D, E and F in (**a**), respectively. (**g**, **h**) HRTEM images of circular enclosed areas B and C in (**a**), respectively. (**i**) The corresponding EDX spectrum of the typical molybdenum nanowall.

Figure 3a gives a low-magnification TEM image of the individual molybdenum nanowall. One can see that the surface of the nanowall is covered with some nanoprotrusions and folded regions and that the thickness is not uniform. We selected five areas from the nanowall to have electron diffraction analysis. Firstly, the area B in the top end of the nanowall is of a single-crystalline structure with little defect, since the selected area diffraction (SAED) patterns are sharp and ordered (Figure 3b). A high-resolution TEM (HRTEM) image of area B shown in Figure 3g reveals that in this area, the nanowall is crystalline, with the mean lattice spacing being 0.22 nm between two adjacent crystalline planes, which is consistent with the {110} planes of the body-centered cubic (bcc) Mo. However, not the whole top end is of a single-crystalline structure. The area C is of a quasi-single-crystalline structure, as one may see from its SAED patterns (Figure 3c). Figure 3h shows the HRTEM image of area C, where the arrowheads point out the existing nanoinclusions. The SAED patterns (Figure 3d,e) of the folded regions of the middle part (areas D and E) show that they are polycrystalline with textured structures, which contain a lot of dislocations and stacking faults. Finally, the SAED pattern f(Figure 3) of area F (the bottom of the nanowall) shows ring patterns of a typical polycrystalline structure. Therefore, the molybdenum nanowalls have a polycrystalline part in the bottom and gradually grow into a crystalline structure at the top end. In addition, the energy-dispersive X-ray spectroscopy (EDX) spectrum of the individual molybdenum nanowall is displayed in Figure 3i. There are Mo, Cu, O, and Cr elements having the atom percentage of about 71.24%, 22.75%, 4.80%, and 1.22%, respectively. Here, the element Cu and Cr may come from the TEM copper grid and the background signal of the lens cone. One may notice that the element O is present in small amounts in the molybdenum nanowall (only 4.80 atom percentage), which probably originates from the amorphous layer on the surface of the individual nanowall (top left in Figure 3g and top right in Figure 3h). Because only very little oxygen exists, we believe that the prepared nanowalls are molybdenum nanowalls rather than molybdenum oxide ones. However, they may have a surface layer of amorphous molybdenum oxide.

**Figure 4:**
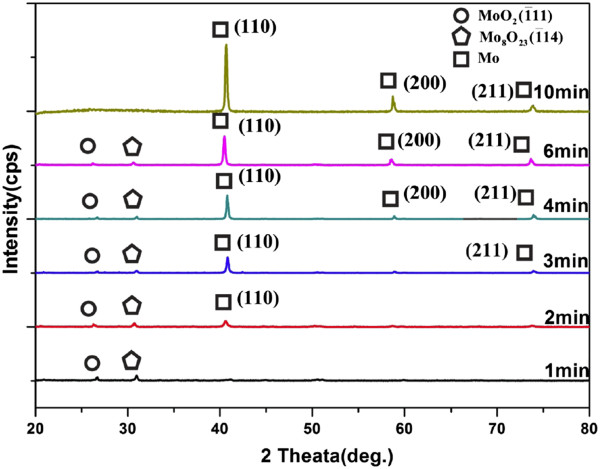
**Typical XRD patterns of the samples prepared with different growth times.** The growth times of the samples are as follows: 1 min, 2 min, 3 min, 4 min, 6 min, and 10 min, respectively.

Further, Figure 4 shows the typical XRD patterns of the samples of the following growth times: 1 min, 2 min, 3 min, 4 min, 6 min, and 10 min, respectively. At the initial stage of growth, there are two peaks indexed to MoO_2_ and Mo_8_O_23_, respectively. With the increase of growth time, new peaks indexed to Mo appear. For 3 min, the peaks of both molybdenum and molybdenum oxides appear. When the growth time is more than 10 min, all the peaks can be indexed to metallic Mo with of a pure bcc structure with a cell constant of *a* = *b* = *c* = 3.15 Å, while the original two peaks indexed to molybdenum oxides disappear. This sequential XRD spectra indicate that from the substrate to the Mo nanowalls, there are nanostructures of Mo oxide in the mid-layer.

**Figure 5:**
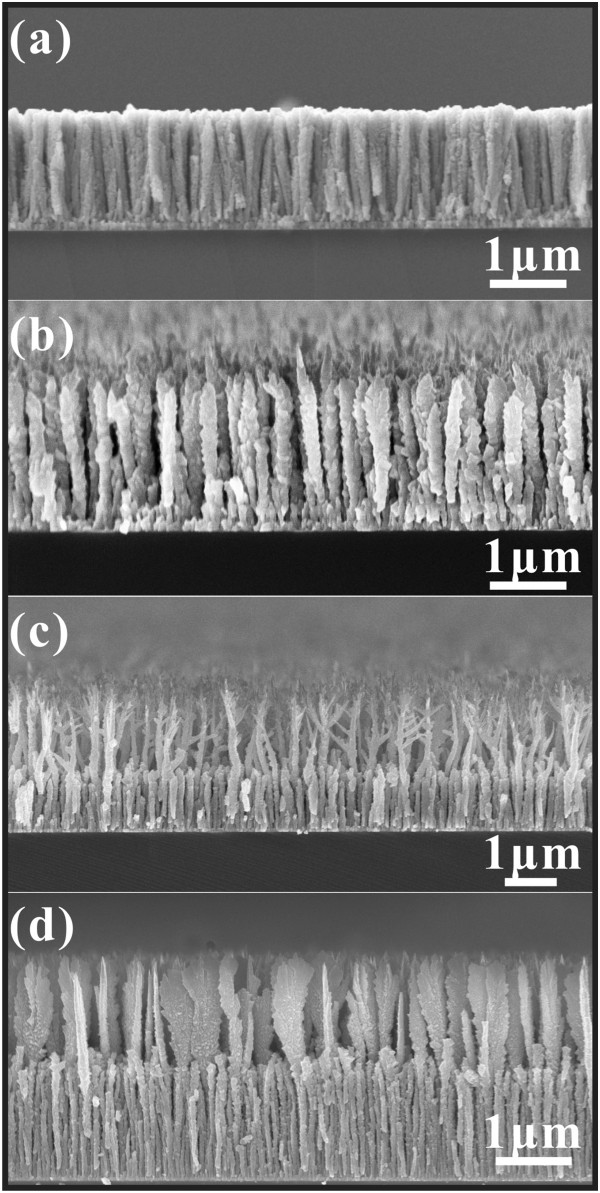
**Side-view SEM images of the samples prepared with different growth times.** The growth times of the samples are as follows: (**a**) 3 min, (**b**) 4 min, (**c**) 6 min, and (**d**) 8 min, respectively.

Now, we shall explain how Mo nanowalls are formed. Our group discussed previously how the molybdenum nanowires were formed [14], and this is the base of our present discussion. To explore the growth mechanism of molybdenum nanowalls, we first need to know the growth process. Figure 5 shows the side-view SEM images of the samples of the following growth times: 3 min, 4 min, 6 min, and 8 min, respectively. One may see that the formation of molybdenum nanowalls may be divided into four stages: uniform and closely aligned nanorods (Figure 5a), nanotrees (Figure 5b and 5c), nanoflakes (Figure 5d), and nanowalls shown in Figure**s**1 and 2. This is the result of the combined effects of atomic arrangement [18], lattice mismatch relationship [19], and competition growth mechanism [14], as explained below. 

**Figure 6:**
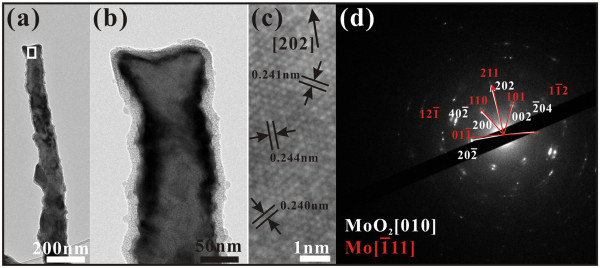
**TEM and HRTEM images and SAED patterns.** (**a**) Typical TEM image of a typical Mo/MoO_2_ nanorod. (**b**) High-magnification TEM image of the top end of the nanorod in (**a**). (**c**) HRTEM image of the rectangular enclosed area in (**a**). (**d**) The corresponding SAED patterns of the nanorod.

We first discuss why in the first growth stage, only nanorods grow. From the HRTEM image (Figure 6c) of the rectangular enclosed area in Figure 6a, the fringe spacings of 0.240 nm, 0.241 nm, and 0.244 nm may be found, corresponding to the lattice spacings of the (200), (002), and (202̄) planes of the monoclinic-structured (Tugarinovite) MoO_2_, respectively. The axial growth plane is (202), and the growth direction is [202]. The SAED patterns of the nanorod were shown in Figure 6d. Both the patterns of monoclinic MoO_2_ and bcc Mo were observed, indicating that the quasi-one-dimensional nanorods are a mixture of Mo and MoO_2_. Some groups [8,9,11,20] have reported that MoO_2_ can act as a seed for the growth of nanorods. The related chemical mechanism has also been reported [11]; some unstable MoO_x_ solid phase that formed may react with the residual oxygen in vapor and then become a stable MoO_2_ crystal. 

**Figure 7:**
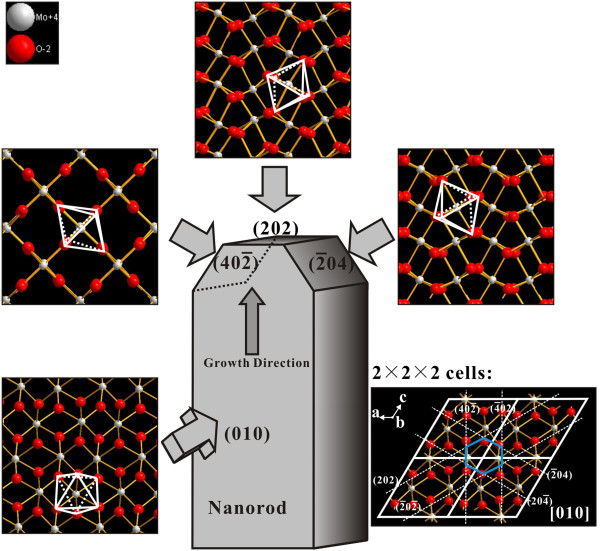
**Structural model of the MoO**_**2**_**nanorod.** The atomic arrangements at the top, side, growth surface, and the 2 × 2 × 2 cells along the [010] zone axis are illustrated. The coordination octahedra of the Mo cations are marked.

It is important to know that a set of the SAED patterns of Figure 6d can be indexed to the [010] zone axis of the monoclinic-structured MoO_2_ crystal. With reference to Figure 7, that means that the normal direction of the wide surface (facing us) of the nanorod corresponds to [010], and the surface plane is (010). As indicated in Figure 7, the (010) plane corresponds to the close-packing plane, and it is not for fast growth. Thus, initially, the [202] direction grows fast to form nanorods.

**Figure 8:**
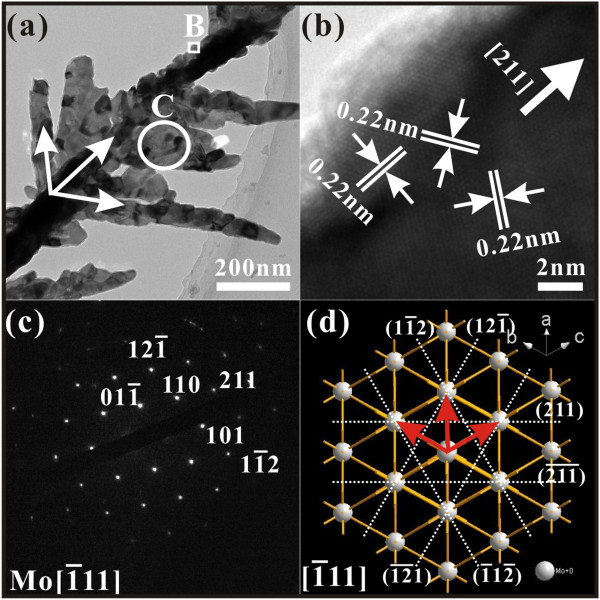
**TEM and HRTEM images, SAED patterns, and atomic arrangements.** (**a**) Typical TEM image of a typical Mo nanotree. (**b**) HRTEM image of the rectangular enclosed area B in (**a**). (**c**) The corresponding SAED patterns of the circular enclosed area C in (**a**). (**d**) Atomic arrangements of the 2 × 2 × 2 cells along the [1̄11] zone axis of bcc Mo.

In the second stage of the growth process, the nanorod grows into a nanotree. Typical TEM images and the corresponding SAED patterns are shown in Figure 8. From Figure 8a, a typical nanotree contains a trunk and branches, with diameters of less than 200 nm and 100 nm, respectively. The branches on both sides of the trunk have a 60° angle. The corresponding HRTEM image (Figure 8b) and the SAED patterns (Figure 8c) are indexed to bcc Mo. No signals of Mo oxides are observed, indicating that the growth in this stage produces metallic molybdenum.

With reference to Figure 7, theoretically, there are two narrow side surfaces existing on both sides of the (202) axial growth plane, and they correspond to potential growth directions of [402̄] and [2̄04]. But how do Mo nanotrees grow from these MoO_2_ surfaces? We attribute this to the fact that the small lattice mismatch relationship between Mo and MoO_2_ crystals contributes to the formation of the nanotree. First of all, when the temperature of the substrates is over 1,428 K, Mo atoms coming from the decomposition reaction now deposit on the substrate instead of MoO_2_. From Figure 6d, one may observe that in the end of the first growth stage, there exists bcc Mo with a [211] growth direction. Also, the [211]_Mo_ and ${\left[202\right]}_{MoO_{z}}$ take the relationship as ${\left[211\right]}_{\text{Mo}}//{\left[202\right]}_{{\text{MoO}}_{z}}$. Therefore, when no MoO_2_ deposits, the original growth direction of MoO_2_ is replaced by the [211] direction of Mo. Actually, [211]_Mo_ and ${\left[202\right]}_{MoO_{z}}$ have a small lattice mismatch relationship. The natural misfit *m* between the two lattices is [19]:

(3) $$m=|d_{\mathrm{Mo}}-d_{MoO_{2}}|/d_{MoO_{2}}$$

Taking MoO_2_ as the reference crystal, the value of *m* between [211]_Mo_ and ${\left[202\right]}_{MoO_{z}}$ is only 7.23%. This explain how the trunk of the molybdenum nanotree grows along the [211] direction of bcc Mo. Furthermore, there exist other two kinds of relationship: ${\left[12\bar{1}\right]}_{\text{Mo}}//{\left[40\bar{2}\right]}_{{\text{MoO}}_{z}}$ and ${\left[1\bar{1}2\right]}_{\text{Mo}}//{\left[\bar{2}04\right]}_{{\text{MoO}}_{z}}$. The corresponding values of misfit are also as small as 8.37% and 8.64%, respectively. For this reason, the condensing Mo grows along the [121̄] and [11̄2] directions as the branches, on both sides of the [211] trunk. The angle between the [211] and [121̄] and the angle between the [211] and [11̄2] are both 60°, which are consistent with the observation from Figure 8a. Here, it should be pointed out that in the bcc structure of Mo, <211 > shows six geometrically equivalent directions (Figure 8d), and in theory, nanowires may grow along all these directions as Zhou et al. [14] suggested. However, we did not observe these in the present work, and this is attributed to the limitation due to the spatial confinement [21].

**Figure 9:**
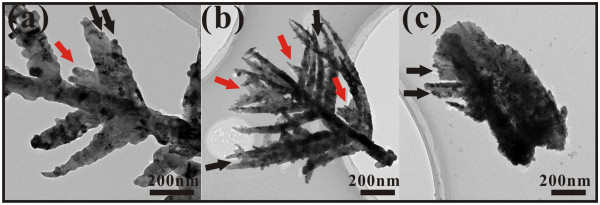
**TEM images of samples in different moments of growth process from molybdenum nanotrees to molybdenum nanoflakes.** (**a**) TEM image of a typical nanotree. (**b**) TEM image of the mid-phase between nanotree and nanoflake. (**c**) TEM image of the original stage of nanoflake. Here, the black arrows point out locations of the main branches, while the red arrows emphasize the new mini-branches.

**Figure 10:**
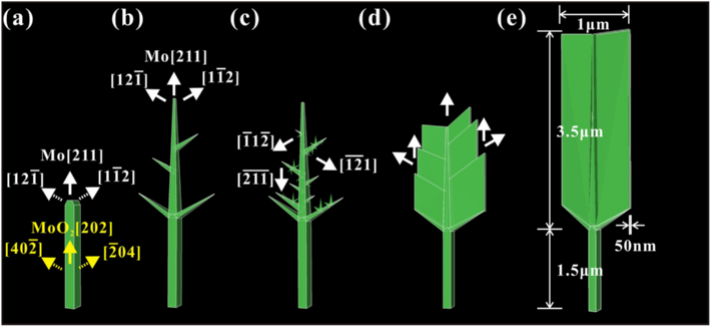
**3D geometrical model of the growth process of molybdenum nanowall from nanorod to nanowall structure.** (**a**) nanorod, (**b**) nanotree, (**c**) nanotree with mini-branches, (**d**) nanoflake, and (**e**) nanowall.

In the third stage, Mo nanowalls start to form from a flake shape. Figure 9 shows TEM images of the samples in different moments of the growth process from the nanotrees to the nanoflakes. When the growth of main branches along the [121̄] and [11̄2] directions is terminated by the adjacent nanotrees, the energies of growth will be redistributed into the following three parts: While the trunks continue to grow, more and more main branches (primary dendrites) can grow out from the sides of the trunk. Furthermore, as emphasized by the red arrowheads, new mini-branches can grow out from the existing branches. As these growth processes continue, two-dimensional flake structures will finally form. The competition growth process [14] plays an important role in this stage as the growth of some branches terminates due to limitation of growth space. However, how the competition growth process here leads to the only two-dimensional growth needs further detailed investigation. The illustration given in Figure 10 may help one to understand the whole growth process. 

**Figure 11:**
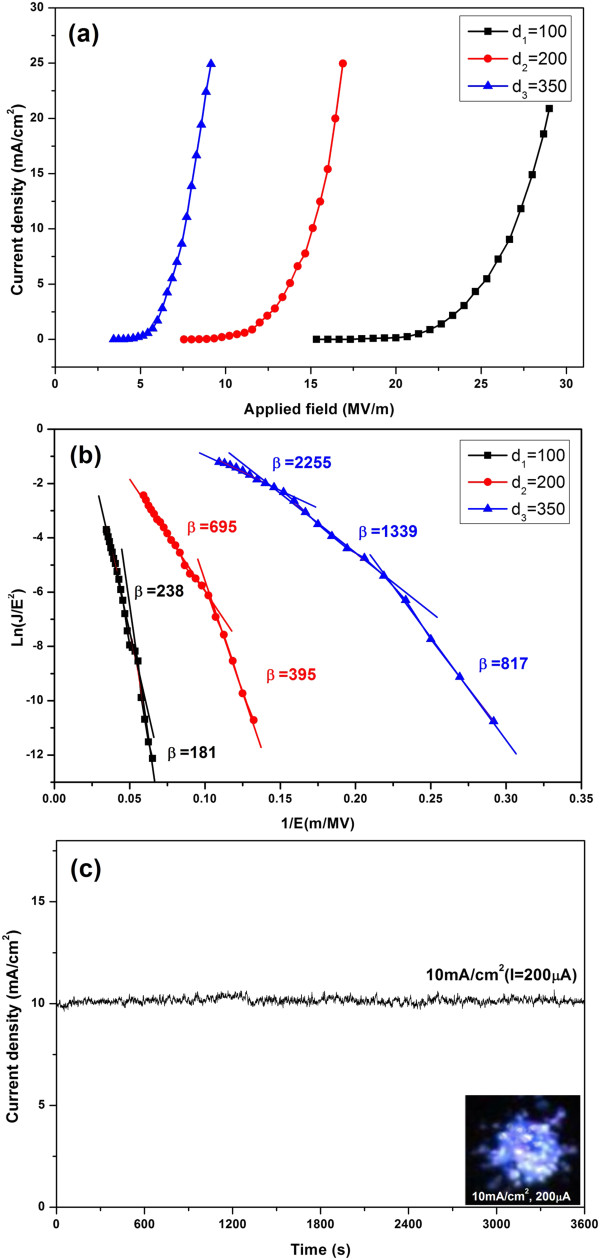
**Field emission properties of Mo nanowall films with an area of 0.02 cm**^**2**^**on stainless steel substrate.** (**a**) The *J*-*E* curves measured at different vacuum gaps (denoted). (**b**) The corresponding F-N plots. (**c**) The emission stability curve of the sample. The inset is the emission site distribution image.

The field emission properties of molybdenum nanowall films were investigated. Here, the molybdenum nanowall films with an area of 0.02 cm^2^ on the stainless steel substrate were studied in an ultra-high vacuum (approximately 2 × 10^−7^ Torr) system by using a transparent anode technique with vacuum gaps of 100, 200, and 350 μm, respectively. After a few cycles of applying an electric field in the gap, the field emission reached a repeatable and stable stage, and the current and voltage data were recorded. Figure 11a shows the curves of field emission current density versus applied field, obtained with the different vacuum gap spacings, revealing that the turn-on field (*E*_to_) and threshold field (*E*_thr_), which are defined as the values of the applied electrical field to induce field emission current densities of 10 μA/cm^2^ and 10 mA/cm^2^, respectively, decrease when the vacuum gap increases from 100 to 350 μm (Table 1). This indicates the significant role the geometrical field enhancement played by the Mo nanowalls. A larger vacuum gap leading to a lower turn-on field was also observed for molybdenum and its oxide nanostructures by others [10,20,22]. The turn-on field of our samples is higher than the best data from other metallic Mo one-dimensional nanostructures [11,14,22] and is due to their smaller aspect ratio (approximately 70). However, this is not hidden in their application in high-current, high-voltage vacuum devices, as may be seen below. Their typical emission current density can reach 25 mA/cm^2^, and the largest current density of 57.5 mA/cm^2^ was recorded from the sample grown on the silicon carbide substrate before vacuum breakdown. 

**Table 1:** **Values of turn-on field and threshold field of molybdenum nanowall films at different vacuum gaps (*d*)**

**Type of materials**	***d*(μm)**	**Turn-on field(V/μm)**	**Threshold field (V/μm)**
Molybdenum nanowall films	100	17	27
	200	8.3	15.1
	350	4.0	7.6

Figure 11b shows the corresponding F-N plots. One can see that these curves may be seen to compose of some discrete regions. One may artificially define the regions so that the regions are nearly straight lines. However, they have different slopes.

The values of field emission (FE) enhancement factor *β* of the molybdenum nanowall films were calculated using the F-N formulation [23]:

(4) $$S_{\mathrm{FN}}=\frac{d\left(\ln\left(J/E^{2}\right)\right)}{d\left(1/E\right)}=-\frac{6.53\times 10^{9}\phi^{3/2}}{\beta }$$

where *Ф* is the work function of the molybdenum nanowall, which adopts the work function (4.3 eV) of bulk molybdenum materials. *S*_FN_ is the slope of the F-N plot. According to Equation 4, we obtained the values of FE enhancement factor *β* for the sample with different vacuum gaps. As shown in Figure 11b, the *β* values in low-field emission regions are calculated, and they are 181, 395, and 817 for different *d* values of 100, 200, and 350 μm, respectively. It is clear that as vacuum gap increases, the *β* value increases. On the other hand, in each curve, the absolute value of the slope at the high-field region is lower than that at the low-field region; thus, the corresponding value of *β* at the high-field region is higher than that at the low-field region. In order to understand what causes the non-linearity in the F-N plots, we consider the following: First, the whole emission process may be affected by the complexity of the material property of the nanowall films. We have known that the molybdenum nanowall films consist of a nonmetallic part. The individual nanowall is covered with a very thin layer of amorphous molybdenum oxide, and there is a layer of nanorods containing molybdenum oxides existing between the nanowalls and the substrates. Both of them may have an effect on the conductivity of the material. When the applied field is very high and, thus, the emission current is very high, the FE enhancement factor *β* would be suppressed because the increase of the voltage drop in the nanowall film itself can result in a decrease of the local field on the surface of emitters. However, the calculated value of *β* at the high-field region is higher, so the above reason does not suit our case. In fact, we will show that the mid-layer and the molybdenum nanowalls both have very good conductivity, while the oxide layer is too thin to have such effect. The higher value of *β* at the high-field region may be explained by both the increasing effect of the thermionic emission and the increasing number of emission sites. Firstly, thermionic emission will gradually increase its effect in the high fields when the current density becomes high. The temperature of the emitters will increase due to the Joule heating. More and more hot electrons will take part in the emission, and thus, the emission will be enhanced. Secondly, the number of emission sites will increase in the high fields. We suggest that the sharp edges of the nanowalls serve as main electron emitters in the low fields, although it is not yet clear without observing the emission pattern of an individual nanowall. At higher fields, however, the nanoprotrusions adhere to the nanowall bodies, and/or the nanorods may be expected to contribute to the total emission current. The increasing emission sites with different values of *β* may enhance the emission, thus leading to the non-linearity in the F-N plots. The detailed explanation awaits to be given after more theoretical and experimental studies.

Figure 11c shows the field emission stability curve, revealing that the molybdenum nanowall films have a relatively stable field emission at a current density of 10 mA/cm^2^ with a fluctuation of about approximately 1.18% in an hour of testing time. The distribution of field electron emission sites was also recorded (bottom right inset in Figure 11c), showing that over 70.01% of the area has bright spots and that the distribution of emission sites is relatively homogeneous, with a value of 66.7%.

Compared with other various emitter materials (listed in Table 2), the turn-on field and threshold field of molybdenum nanowalls are higher than C nanotubes [24], SiC nanowires [25], tungsten oxide nanowires [26,27], and some main molybdenum and its oxide one-dimensional nanostructures, i.e., MoO_2_ nanowires [11], MoO_3_ nanowires [11,13], Mo nanowires [11,14], MoO_3_ microbelts [15,16], and pinaster-like MoO_2_ nanoarrays [8]; however, they are lower than other materials such as Cu_2_S nanowires [28], ZnO nanowires [29], and other nanostructures of molybdenum oxides, i.e., MoO_2_ nanostars [9], Mo oxide nanowires with nanoprotrusions [20], MoO_2_ nanorods [7], and MoO_3_ nanoflowers [30]. In addition, the value of a stable large current density of about 57.5 mA/cm^2^ recorded so far is higher than most of the emitter materials reported except for C nanotubes [24] and MoO_3_ nanobelts [10], which shows the attractive perspective of the molybdenum nanowalls for the high-current density application demanded in a number of vacuum devices, such as X-ray source and microwave source. Moreover, the current stability is better than most of the materials, especially the molybdenum and its oxide one-dimensional nanostructures [8,9,11,13,14,16,29]. 

**Table 2:** The field emission properties of various emitter materials

**Material**	***E***_**turn-on**_**(V/μm)**	***E***_**threshold**_**(V/μm)**	**Reported largest current density (mA/cm**^**2**^**)**	**Fluctuation of emission current density**
Mo nanowalls	4.0	7.6	57.5	1.18%, 10 mA/cm^2^
C nanotubes [24]	<0.75	1.6	3,000	
SiC nanowires [25]	0.9	2.5 to 3.5	18	3%, 5 mA/cm^2^
Cu_2_S nanowires [28]	6.0	12	0.6	2%, 0.4 mA/cm^2^
ZnO nanowires [29]	>6.0	>11	2.0	
Mo nanowires [11,14]	2.2	6.24	18	5%, 10 mA/cm^2^
MoO_2_ nanowires [11]	2.4	5.6	16	2.5%, 10 mA/cm^2^
MoO_3_ nanowires [11,13]	3.5	7.65	13	10%, 10 mA/cm^2^
MoO_2_ nanorods [7]	4 to 18			
Pinaster-like MoO_2_ nanoarrays [8]	2.39		5.2	9.2%, 2.8 mA/cm^2^
MoO_3_ nanobelts [10]	8.7	12.9	1,000	15%, 260 mA/cm^2^
MoO_3_ microbelts [15,16]	2.6	6.4	14	2%, 0.94 mA/cm^2^
MoO_3_ nanoflowers [30]	4.3		0.14	10%, 0.01 mA/cm^2^
Mo oxide nanostars [9]	11.3		1.36	13%, 0.35 mA/cm^2^
WO_2_ nanowires [26]	1.357	2.38	12.8	
WO_3_ nanowires [26]	1.72	2.87	15	
W_18_O_49_ nanowires [27]	2.0	4.37	14	2%, 0.16 mA/cm^2^

**Figure 12:**
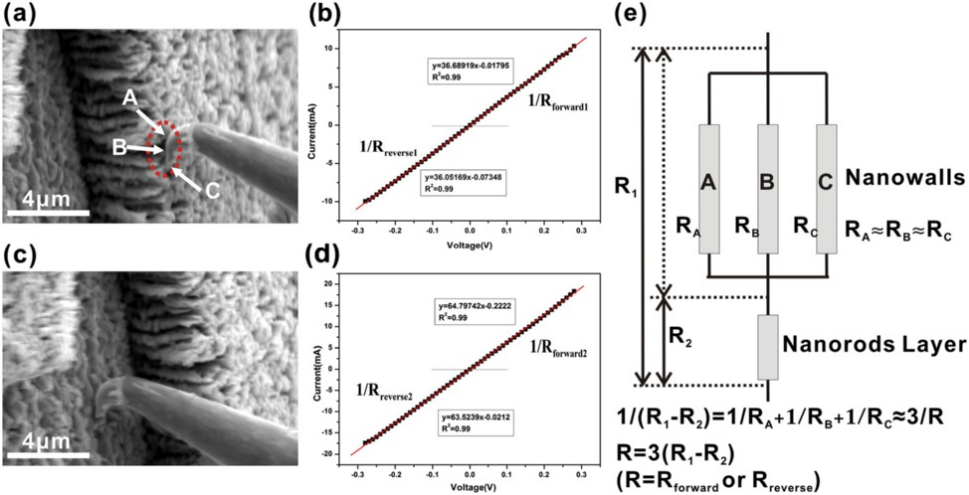
**SEM images and corresponding *I *-*V *characteristics and schematic circuit diagram.** (**a**) SEM image showing the W microprobe in contact with the top end of three single nanowalls for electrical conductivity measurements. (**b**) The corresponding *I*-*V* characteristic curve in (**a**). (**c**) SEM image showing the W microprobe in contact with the mid-layer for electrical conductivity measurements. (**d**) The corresponding *I*-*V* characteristic curve in (**c**). (**e**) The corresponding schematic circuit diagram.

The electrical transport properties of the nanowall films were also investigated. Figure 12 gives the SEM images showing the tungsten (W) microprobe in touch with the sample surface for electrical conductivity measurements and the conduction *I*-*V* characteristics. First, the W microprobe contacted the top surfaces of the nanowalls and touched only the top ends of three nanowalls in the measurement (Figure 12a). Sweeping voltage was applied. From Figure 12b, the resistance for forward applied voltage (*R*_forward 1_) and the resistance for reverse applied voltage (*R*_reverse 1_) are 27.26 and 27.74 Ω, respectively, which were calculated by fitting the *I*-*V* plots. Second, the W microprobe contacted the surface of the mid-layer (Figure 12c). Similarly, the resistance for forward applied voltage (*R*_forward 2_) and the resistance for reverse applied voltage (*R*_reverse 2_) were obtained by fitting the *I*-*V* plots from Figure 12d. The corresponding values are 15.43 and 15.74 Ω, respectively. Considering the fact that the measured three nanowalls A, B, and C are side-by-side and have nearly the same size, we assume that the resistances *R*_A_, *R*_B_, and *R*_c_ are parallel connected and approximately equal. The resistance of a typical nanowall may be estimated through the following: ${\text{R}}_{\text{forward}}=3\times \left({\text{R}}_{\text{forward}\;1}-{\text{R}}_{\text{forward}\;2}\right)=35.49\Omega ;{\text{and R}}_{\text{reverse}}=3\times \left({\text{R}}_{\text{reverse}\;1}-{\text{R}}_{\text{reverse}\;2}\right)=36\Omega$, according to the schematic circuit diagram shown in Figure 12e. Here, the resistance of the mid-layer and the contact resistance between the mid-layer and the substrate have been eliminated. However, the contact resistance between the W microprobe and nanowall cannot be completely eliminated since the contact in each measurement is different. Because the resistance for forward applied voltage is nearly equal to the resistance for reverse applied voltage, we believe that such difference is small. Furthermore, the shape of the *I*-*V* curves is almost linear, suggesting that there were good Ohmic contacts existing between the nanowalls and the mid-layer, between the mid-layer and the substrate, and between the W microprobe and the contacting surfaces.

Accordingly, the conductivity of the single molybdenum nanowall may be estimated using the equation below:

(5) $$\sigma =h/\left(Rwt\right)$$

where *R* is the resistance, while *h*, *w*, and *t* are the height, width and thickness of a single nanowall, respectively. Taking the average values of a height of 3.5 μm, width of 1 μm, thickness of 50 nm, and *R* equal to 35.49 and 36 Ω, we can obtain the conductivity of a single molybdenum nanowall: $\sigma_{\text{forward}}=1.97\times 10^{4}\Omega^{-1}{\text{cm}}^{-1};\text{and}\;\sigma_{\text{reverse}}=1.94\times 10^{4}\Omega^{-1}{\text{cm}}^{-1}$. Table 3 lists the calculated conductivities of molybdenum nanowall samples grown on different substrates, showing that these values, ranging from 0.88 × 10^4^ Ω^−1^ cm^−1^ to 5.23 × 10^4^ Ω^−1^ cm^−1^, are usually about one to four orders of magnitude higher than those of other reported nanostructures [6,26,28,31-34]. We should like to note that most of the conductivity values of the Mo nanowall on different substrates are about an order of magnitude less than the value of the molybdenum bulk material (approximately 19.46 × 10^4^ Ω^−1^ cm^−1^) [35]. Such high conductivities explain why molybdenum nanowalls can sustain a stable high field emission current density. This outstanding electrical transport property of the molybdenum nanowall is mainly due to its metallic intrinsic property and could be related to the 2D wall-like structure. Beyond doubt, Mo nanowalls with their excellent electric conductivity is favorable to the high-current and high-current density application in vacuum electron devices. 

**Table 3:** The calculated conductivities of different individual molybdenum nanowalls compared with other nanomaterials

**Material**	**Substrate**	**Conductivity (×10**^**4**^**Ω**^**−1**^ **cm**^**−1**^**)**
Molybdenum Nanowalls	Silicon carbide	0.88
	Stainless steel	5.23
	Silicon 100	4.6
	Silicon 111	1.97
Molybdenum bulk material [35]		19.46
Agave-like 1D ZnO nanostructures [6]		0.337
Boron nanotubes [31]		0.004
CuO nanowires [32]		0.00078
Cu_2_S nanowires [28]		0.001
WO_2_ nanowires [26]		0.00206
WO_3_ nanowires [26]		0.00097
W_18_O_49_ nanowires [33]		0.000258
1D molybdenum nanotips [34]		0.00507

## Conclusions

Vertically aligned molybdenum nanowalls can be prepared on varied substrates by thermal vapor deposition. Their formation undergoes through a four-stage process associated with the production of molybdenum and its dioxide nanorods, molybdenum nanotrees, molybdenum nanoflakes, and molybdenum nanowalls. Atomic arrangement, lattice mismatch relationship, and competition growth all together contribute to the growth mechanism. The nanowalls can sustain a very much larger field emission current density and better emission stability as compared to most of other nanostructures. Moreover, the single nanowall is shown to have a remarkably high electrical conductivity. The results suggest that molybdenum nanowalls should have promising futures in the application of vacuum electron devices that demand both high current and high current density.

## Competing interests

The authors declare that they have no competing interests.

## Authors' contributions

YS carried out all the experimental processes, characterization, and mechanism research. SZD, YZ, FL, JC, and NSX conceived the study and participated in its design and coordination. YS and NSX drafted the manuscript. All authors read and approved the final manuscript.

## Author details

^1^State Key Laboratory of Optoelectronic Materials and Technologies, Sun Yatsen University, Guangzhou 510275, People's Republic of China. ^2^Guangdong Province Key Laboratory of Display Material and Technology, Sun Yat-sen University, Guangzhou 510275, People's Republic of China. ^3^School of Physics and Engineering, Sun Yat-sen University, Guangzhou 510275, People's Republic of China.
